# Comparison of Seafood Consumption in a Group of Italian Mother-Child Pairs

**DOI:** 10.3329/jhpn.v31i4.20000

**Published:** 2013-12

**Authors:** Laura Deroma, Francesca Valent, Maria Parpinel, Fabio Barbone

**Affiliations:** ^1^Regional Coordinator Centre for Rare Diseases, University Hospital “Santa Maria della Misericordia”, Udine, Italy; ^2^Regional Health Directorate, Friuli Venezia Giulia Region. Udine, Italy; ^3^Unit of Hygiene and Epidemiology, Department of Medical and Biological Sciences, University of Udine. Udine, Italy; ^4^Institute of Hygiene and Clinical Epidemiology, University Hospital “Santa Maria della Misericordia”, Udine, Italy, Unit of Hygiene and Epidemiology, Department of Medical and Biological Sciences, University of Udine, Udine, Italy

**Keywords:** Child, Food habits, Mothers, Pregnancy, Seafood, Italy

## Abstract

Seafood is an important component of healthful human diets. Intake of seafood is recommended both for young women and children. In fact, it is a good source of high-quality protein, low in saturated fats, and rich in essential nutrients (e.g. iodine, iron, choline, and selenium) and long-chain polyunsaturated fatty acids (LCPUFAs), especially omega-3. However, the relationship between maternal diet and the children's dietary habits is controversial. This study investigated the possible association between the seafood consumption by mothers and that by their 8-11 years old children and compared maternal seafood intakes during pregnancy and about 10 years later. The seafood consumption by 37 pregnant women was assessed in 1999-2001. In 2009, mothers were asked to report their weekly intake and their children's. Mother-child pairs showed a similar consumption pattern: the overall intake was 1.28±0.77 vs 1.19±0.64 (p=0.49) while the sum of specific items was 3.71±3.01 vs 3.18±2.90 (p=0.049). However, it cannot be discerned whether maternal diet affected the children's nutritional habits or vice-versa. In fact, mothers showed to have a higher seafood intake about 10 years after pregnancy (3.71 vs 1.83; p<0.001), suggesting that a progressive modification of dietary habits occurred after delivery, possibly due to the influence of maternal diet on the nutritional habits of offspring or due to the presence of children in the family unit, that could have influenced maternal dietary habits. This dietary improvement could be brought forward through educational interventions addressed to young women, that could also allow a more informed choice of the healthier species of fish both for them and their children.

## INTRODUCTION

Seafood is an important component of healthful human diets since it is a good source of high-quality protein, low in saturated fats and rich in essential nutrients (1), such as iodine, iron, choline, and selenium (2,3). Moreover, seafood is rich in long-chain polyunsaturated fatty acids (LCPUFAs), especially omega-3 (eicosahexaenoic acid and docosahexaenoic acid) (4), fish being the main source in the Western diet (5).

Nevertheless, seafood may also contain persistent organic pollutants, such as dioxins and polychlorinated biphenyls (PCBs) (1) and represents the main source of mercury in humans (6). Both contaminants and LCPUFA levels have a considerable variability, being affected by seafood species, size, location of harvest, age, and composition of feed. Fish at high food-chain levels, particularly large predatory species, have higher LCPUFAs and mercury content (4) and lipophilic compounds, such as dioxins and PCBs found in the fatty tissue of some fish. Moreover, higher LCPUFA levels are found in fatty and ocean fish (1).

Considering the above, similar recommendations were made for young women and children up to 12 years of age, suggesting that both groups “may benefit from consuming seafood, especially those with relatively higher concentrations of EPA and DHA” but “should avoid large predatory fish” (1).

However, while many studies investigated the association between fish consumption in pregnancy and neurodevelopment in child (7-11), only a few have been conducted to assess fish consumption by children. Moreover, scientific literature provides inconsistent results about the possible association between maternal diet and the children's dietary habits.

A study performed in Scotland on 36 mothers and their children (5.5 to 8.5 years of age) showed that fish intakes by mother and child are not correlated (rs=0.15; p=0.4), and mothers have a higher fish consumption (p=0.02) (12). On the contrary, a randomized telephone survey conducted in the USA showed that more than 80% of mother-child pairs had similar fish intakes (13). Moreover, a qualitative study conducted in Perth (Australia) demonstrated that the frequency of fish consumption within the family unit is largely influenced by the presence of children and their age (14). Likewise, a Finnish study showed some familial dependence between dietary clusters of mother-child pairs of 6 years old children (p=0.035) and reported that a high proportion of children belonging to the “healthy, low-fat” cluster had mothers from the “fat conscious” and “modern, healthy” clusters (15). On the other hand, another study conducted in the United Kingdom on 413 mother-child pairs showed similar intakes of vegetables and fruits but not fish (16).

Even when an association is found, its direction is difficult to ascertain. This is an issue, especially in cross-sectional studies, when data are collected for both mothers and children at the same time but also when data on maternal nutritional habits are collected at different times, such as in the ALSPAC study (17). In fact, whether maternal diet influences the children's nutritional habits or vice-versa still remains an open question.

The main aim of this study was to evaluate whether seafood consumption of 8 to 11 years old children is associated with that of their mothers. A secondary objective was to compare seafood intakes by mothers during pregnancy and about 10 years later.

## MATERIALS AND METHODS

A cohort of 242 mother-child pairs was enrolled between 1999 and 2001 (T1) in Friuli Venezia Giulia region, Northeastern Italy, to evaluate whether prenatal and postnatal exposure to mercury and seafood (particularly through consumption of fish) had an effect on the neurodevelopment in child (18-21). A food frequency questionnaire (FFQ) was administered to mothers by trained personnel about 3 months after delivery, and their general dietary habits during the whole pregnancy period were recorded. The seafood consumption was reported as open-ended responses to three summary questions on the intake of ‘fish’, ‘crustaceans’ and ‘molluscs’, and ‘tuna, mackerel, and sardines in oil’.

Between 2007 and 2009, 154 mothers accepted to participate in the follow-up and their children being tested for neurodevelopmental parameters (21).

In the second half of 2009 (T2), a subsample of 43 mother-child pairs was randomly selected from this cohort in order to describe the seafood intake of both mothers and children. A detailed protocol was arranged, and a trained professional interviewer collected data accordingly after several test interviews. Mother-child pairs were contacted, and a digital audio-recording of each phone call was made to check adherence to protocol (the answers of interviewed subjects were not recorded).

Seven questions on ‘fish’ (boiled, grilled, baked, fried), ‘molluscs’ (boiled, grilled, baked, fried), ‘crustaceans’ (boiled, grilled, baked, fried) and ‘fish in oil’ (tuna, mackerel, sardines), and an additional question on ‘other canned fish’ were administered to mothers through telephone, asking them to report the current seafood consumption of both themselves and their children. An open-ended question on the number of seafood servings consumed per week (summary question) and several questions about the comparison of seafood consumption of mothers (present vs in pregnancy) and mother-child pairs (present) were also included.

A detailed description of the tools used in gathering information on seafood intake at T1 and T2 is reported in Table 1.

### Statistical analysis

The sample-size was chosen to detect a minimum difference of one serving/week (paired data) in seafood intake by mothers and their children at T2. Assuming a standard deviation of the difference of 2, with a significance level of 0.05, and a power of 0.80, a random sample of at least 33 mother-child pairs was required.

Current seafood consumption (T2) for both mothers and children is described as number of servings per week (serving-sizes are reported in Table 1). This was obtained by assigning a weight to each frequency of consumption category (never: 0; less than once a month: 0.02; 1-3 times per month: 0.07; once a week: 0.14; 2-4 times per week: 0.43; 5-6 times per week: 0.79; once a day: 1.00; 2-3 times a day: 2.50; more than 3 times a day: 4.50), as suggested by Willett (22).

**Table 1.** Instruments used in assessing seafood consumption

Time	T1	T2
Administration	1999-2001	Summer-Fall 2009
	Administered face-to-face at the mother's home	Administered through telephone
Study subject	Mother in pregnancy	Mother, 10 years after delivery Child, 10 years of age
Items	(a) Fish	(a) Fish, boiled, grilled, baked
	(b) Crustaceans and molluscs	(b) Fish, fried
	(c) Tuna, mackerel, and sardines in oil	(c) Molluscs, boiled, grilled, baked (d) Molluscs, fried
		(e) Crustaceans, boiled, grilled, baked
		(f) Crustaceans, fried
		(g) Tuna, mackerel, and sardines in oil
		(h) Other canned fish
		(i) Seafood (summary variable)
Serving-size	Fish: 150 g	Fish: 150 g
	Crustaceans and	Molluscs: 150 g
	molluscs: 150 g	Crustaceans: 150 g
	Tuna, mackerel, and	Tuna, mackerel, and sardines in oil: 80 g
	sardines in oil: 80 g	Other canned fish: 1 can
Frequency of consumption	Open. Servings per day or week or month or in pregnancy (unit to be chosen by the mother)	*Items (a) to (h)* Nine possible number of servings (never; less than once a month; 1-3 times per month; once a week; 2-4 times per week; 5-6 times per week; once a day; 2-3 times a day; more than 3 times a day
		*Item (i)*
		Open. Servings per week
Other questions on seafood		Mother: Comparison of present consumption vs that in pregnancy Mother-child pairs: Comparison of present consumption

**Table 2.** Mean weekly seafood consumption by mothers and children at T2 (servings/week)

Type of food	Mother (n=37) Mean±SD	Child (n=37) Mean±SD	p*
Fish			
Boiled, grilled, baked	1.04±1.01	0.97±0.88	0.94
Fried	0.56±0.78	0.47±0.54	0.52
Crustaceans			
Boiled, grilled, baked	0.24±0.27	0.14±0.26	<0.001
Fried	0.06±0.12	0.07±0.19	0.91
Molluscs			
Boiled, grilled, baked	0.46±0.37	0.39±0.39	0.11
Fried	0.22±0.25	0.16±0.24	0.10
Canned fish			
Tuna, mackerel, sardines in oil	0.90±1.00	0.82±1.13	0.12
Other canned fish	0.23±0.55	0.18±0.53	0.47
Seafood			
Aggregate variable (sum of single items)	3.71±3.01	3.18±2.90	0.049
Summary variable	1.28±0.77	1.19±0.64	0.49

*Signed-rank test for paired data

To allow for comparison of maternal seafood intake in pregnancy and at follow-up, the original continuous variables on seafood consumption collected at T1 were converted into categorical variables (nine categories, the same as T2). Moreover, the original seven variables on seafood consumption at T2 were converted into three variables (fish; crustaceans, and molluscs; tuna, mackerel, and sardines in oil), regardless of the cooking type (i.e. fish=boiled/grilled/baked+fried). The number of servings was calculated using the weights described above.

Fisher's exact test was used for comparing proportions while the Signed-rank test was used for comparing means of paired data since all the variables were not normally distributed according to the Shapiro-Wilk test.

The variable ‘season of administration’ was recoded according to astronomical seasons (summer from 21 June to 22 September; autumn from 23 September to 20 December).

All the analyses were performed using SAS software (version 9.1) of the SAS System for Windows (Copyright © 2002-2003 SAS Institute Inc., Cary, NC, USA).

## RESULTS

In September-November 2009, 43 mother-child pairs were called on the phone, and 37 of them were reached and accepted to participate. In 6 cases, the contact was not possible. The information on seafood consumption for the mother-child pairs was collected in late summer (n=10) and autumn (n=27).

### Comparison between seafood intake by mothers and their 8-11 years old children

Table 2 shows the weekly mean consumption of seafood by mother-child pairs. Similar intakes were observed for all items, except boiled/grilled/baked crustaceans, whereof mothers had a higher consumption (0.24±0.27 vs 0.14±0.26 servings/week; p=0.0005).

The mean seafood consumption by mothers was 3.71±3.01 servings/week when the sum of single items (aggregate variable) was considered while it decreased to 1.28±0.77 when analyzing the summary variable, with a mean difference of 2.42±2.59 servings/week (p<0.0001). Similarly, the mean consumption of seafood by children was 3.18±2.90 servings/week when considering the aggregate variable while it decreased to 1.19±0.64 according to the summary variable, with a mean difference of 1.99±2.47 servings/week (p<0.0001).

The comparison between seafood consumption by mothers and children showed that every week mothers ate, on average, half a portion more than their children (3.71±3.01 vs 3.18±2.90; p=0.049) when considering the aggregate variable while no difference was detected when using the summary variables (1.28±0.77 vs 1.19±0.64; p=0.49). In fact, overestimation of the aggregate variable was higher in mothers (2.42±2.59 vs 1.99±2.47; p=0.03).

When specifically asked, 14 mothers (37.8%) declared that their seafood consumption is different from that of their children. In particular, 6 (42.9%) reported to eat less seafood than their children, and 4 of them attributed the difference to the fact that children usually eat several meals at school or at grandparents’ house. On the other hand, 8 mothers (57.1%) reported a higher intake, mostly because their children disliked seafood (n=7).

### Comparison between maternal seafood intake in pregnancy and 10 years later

Table 3 reports the seafood intakes by mothers during pregnancy (using both original open-ended response and its transformation into a closed-ended response) and about 10 years later. When using closed-ended responses, current seafood consumption resulted in slightly overestimation (overall seafood consumption: 1.58 vs 1.83; p<0.001) but was, on average, much higher than in pregnancy (3.71 vs 1.83; p<0.001), with relevant differences for all items (Table 3).

However, when specifically asked, only 10 mothers reported their current seafood consumption as higher than in pregnancy while others reported similar (n=15) or even lower consumption (n=10). Two mothers were not able to answer.

## DISCUSSION

The main aim of this study was to compare seafood consumption in a group of Italian mothers and their 8-11 years old children.

Similar intakes were reported by mothers for fish, molluscs, canned fish, and fried crustaceans while a difference was only observed for boiled/grilled/baked crustaceans that are more consumed by mothers, although consumption is quite modest (0.24 vs 0.14 servings/week). On the whole, when aggregate variables were used (defined as a sum of single items), a slightly higher seafood intake was assessed for mothers (3.71 vs 3.18; p=0.049) while the comparison between summary variables, expression of the overall seafood intake as directly reported by mothers, yielded no differences, the mean consumption being about 1 serving/week both for mothers and children (1.28 vs 1.19; p=0.49). Consistently, 62.2% of the mothers declared to have a seafood consumption comparable to that of their children, and possible differences may be due to the child's personal taste or can arise when meals are not consumed at home. These results are in contrast with those from several studies reporting different intakes by mothers and their children (12,16) but in line with the similar intakes reported by Imm *et al.* (13) and with the strong correlations between the macronutrient and energy intakes of mother-child pairs showed in the ALSPAC study (17). Moreover, these are consistent with the evidence provided by Ovaskainen *et al.* who reported an association between dietary patterns in mother-child pairs (15). However, it is difficult to predict whether maternal diet influenced the child's habits, or the opposite occurred. As suggested by Brion *et al.*, maternal diet could have affected the offspring's diet both during pregnancy via intrauterine mechanisms, and after delivery, due to a direct influence of dietary habits (17). Nevertheless, McManus *et al.* showed that the presence of children strongly affects the dietary habits of the family unit (14); thus, the direction of this association remains an issue.

**Table 3.** Mean weekly seafood consumption (servings/week) by mothers during pregnancy (T1) and 10 years later (T2)

Type of food	Seafood consumption in pregnancy (T1) (Servings/week)			Seafood consumption at follow-up (T2) (Servings/week)	
	Open-ended response^a^ (Mean±SD)	Closed-ended response^b^ (Mean±SD)	P^c^	Closed-ended response (Mean±SD)	P^d^
Fish (n=37)	0.70±0.65	0.80±0.76	0.12	1.60±1.59	<0.001
Molluscs and crustaceans (n=37)	0.27±0.40	0.32±0.39	0.003	0.98±0.71	<0.001
Tuna, mackerel, sardines in oil (n=37)	0.60±0.63	0.71±0.75	<0.001	0.90±1.00	0.04
Other canned fish (n=37)	-	-	-	1.12±1.45	0.006^e^
Overall seafood consumption (n=37)	1.58±1.01	1.83±1.09	<0.001	3.71±3.01	<0.001

^a^ Original response;

^b^ Transformed response;

^c^ Signed-rank test for paired data, comparison of consumption in pregnancy: open-ended vs closed-ended responses;

^d^ Signed-rank test for paired data, comparison of consumption in pregnancy vs at follow-up, according to closed-ended responses;

^e^ Comparison between consumption of ‘tuna, mackerel, sardines in oil’ during pregnancy and consumption of ‘tuna, mackerel, sardines in oil’ and ‘other canned fish’ at follow-up

In any case, although the mother-child comparisons did not yield remarkably different results when summary or aggregate variables were considered, large differences were seen in absolute values both for mothers and children. These were expected since it is well-known that, as the number of items increases, overestimation increases as well (23). Moreover, a pilot study conducted by Cavan *et al.* to test a questionnaire on fish consumption already demonstrated that aggregate variables provide higher estimates than summary variables (24), and similar results were found by Mina *et al.* regarding fresh fish (25).

The secondary aim of this study concerned the seafood consumption by mothers. Maternal seafood intake at follow-up was much higher than in pregnancy, both overall (3.71 vs 1.83 servings/week) and when single items were considered. Nevertheless, 15 mothers (40.5%) declared similar intakes, when specifically asked, and 10 reported higher or lower intakes, and only 2 were not able to answer the question. In spite of this, a true difference is likely since its magnitude is too large to be explained by methodological issues alone, such as the difference in the administration of interview (face-to-face vs on the phone) and collection tools (open-ended vs closed-ended questions; 3 summary questions vs 8 more specific questions) between T1 and T2. Moreover, the apparent inconsistency between quantitative estimates and a specific categorical question could be explained by the difficult recall of past dietary habits, especially when the time lag is long (7-9 years). In fact, a different intake could have several different explanations, including the possible change in several factors, such as seafood availability or price, or socioeconomic improvement. Moreover, seafood intake could have been lowered in pregnancy due to the consciousness of the relationship among seafood, mercury, and neurodevelopment. However, this is not very likely since specific information campaigns were not conducted in Italy until the late 1990s, and the effect of public health and safety information campaigns is controversial (26-29). Another hypothesis is that mothers didn't actually report their current ‘usual’ consumption but their ‘current’ consumption, this being affected by seasonal variations. However, a more reasonable explanation is that dietary habits, especially fish consumption, have progressively modified through the years within the family unit, possibly due to the strong influence of the presence of children (14). To our knowledge, only in the ALSPAC study, data on maternal nutritional habits were collected both during pregnancy and at a later stage (about 4 years after delivery), and positive fair correlations were found when intra-individual comparisons were performed (17). However, since the aim of the paper by Brion *et al.* was to evaluate macronutrients and energy intake, information on dietary patterns or specific food was not reported, and a comparison is not feasible (17).

The main strength of this study is that data on mothers and children were collected at the same time (T2), using the same tool, administered through phone by trained professionals, thus allowing for a comparison free from information bias. Moreover, the questionnaire used at T2 was derived from a larger one, validated on 7 day dietary records (30). Finally, although FFQs provide a higher overestimation than dietary records (31) when the total energy estimates are compared with those obtained using the doubly-labelled water method (32), an FFQ is less demanding in terms of compilation, interpretation, and data imputation; and its ease of administration and cheapness make it the most suitable method to collect information on usual food and nutrient intake so far (22).

On the other hand, information on children was reported by mothers. Parents usually provide reliable information on food consumed at home but may not be informed about food consumed by their children when they are not at home; thus, a correct estimate of food intake also depends on the interest, motivation, and memory precision not only of mothers but also of other caregivers who indirectly collaborate in compiling the questionnaire (32). Moreover, the accuracy of dietary information may be compromised by a bias towards underestimation for obese children with at least one obese parent (33). Considering all these, we decided to interview mothers because the ability of reporting food consumption is limited in children younger than 12 years (32).

### Conclusions

Mothers and their 8-11 years old children have essentially a similar seafood intake but it cannot be discerned whether maternal diet affected the children's nutritional habits or vice-versa. In fact, mothers showed to have a higher seafood intake about 10 years after pregnancy, suggesting that a progressive modification of dietary habits occurred after delivery. This dietary improvement could be brought forward through educational interventions addressed to young women. These should receive a correct information on both nutritional content and the possible contamination of fish and seafood in general, to be able to choose the healthier species both for them and their children.

## ACKNOWLEDGEMENTS

The authors would like to thank Claudia Carosi who performed the interviews at T2 and all the mothers and children who took part in this study.
